# Portable, precise, and RNA extraction-free: RT-RAA technology for rapid early RSV identification and prevention

**DOI:** 10.3389/fcimb.2025.1640503

**Published:** 2025-09-02

**Authors:** Zhenfei Wang, Jing Zhang, Xiao He, Chunming Wang, Zhenyue Li, Zitong Yang, Cheng Zhang, Tingli Fan, Kai Su

**Affiliations:** ^1^ Institute of EcoHealth, School of Public Health, Cheeloo College of Medicine, Shandong University, Jinan, Shandong, China; ^2^ Department of Agricultural and Animal Husbandry Engineering, Cangzhou Technical College, Cangzhou, China; ^3^ College of Veterinary Medicine, Hebei Agricultural University, Baoding, China

**Keywords:** respiratory syncytial virus, real-time reverse transcription recombinase-aided amplification, visual detection, rapid detection, RNA extraction-free

## Abstract

The Respiratory Syncytial Virus (RSV) is a significant agent linked to respiratory infections, representing a considerable health risk for vulnerable populations, including infants, older adults, and those with weakened immune systems. This research successfully introduces an RNA extraction-free rapid detection technique for RSV utilizing real-time reverse transcription recombinase-aided amplification (RT-RAA) technology. Through the crafting of specific primers and probes, this approach enables precise identification of RSV without any interference from other prevalent respiratory viruses. Tests for sensitivity indicated that the detection threshold at a 95% confidence interval was 159 copies per reaction, while the visual detection limit was found to be 1,177 copies per reaction. Testing on clinical samples demonstrated a high degree of consistency with reverse transcription quantitative real-time PCR (RT-qPCR), achieving a Kappa value of 1, which signifies excellent correlation. Furthermore, the amplified products from RT-RAA can be seen with the aid of a portable blue light device, rendering this method appropriate for rapid detection in settings where resources are limited. A total of 265 clinical samples were tested, and the results showed 100% concordance with RT-qPCR. Compared with rapid antigen detection tests (RADTs), RT-RAA exhibited significantly higher sensitivity (100% *vs*. 93.8%). The rapid detection method for RSV using RT-RAA offers solid technical assistance for the early identification and prevention of RSV.

## 1 Introduction

Following the COVID-19 pandemic, interest in virology has notably increased, particularly in pathogens that were previously overlooked but have significant impacts on human health. One such pathogen is respiratory syncytial virus (RSV), which significantly contributes to respiratory infections and is the primary viral agent accountable for acute lower respiratory tract infections (ALRTIs) in children younger than five years old (Shi et al., 2017; Bénet et al., 2017). Due to its high transmissibility and associated mortality, infection with RSV has become a major public health issue (Langedijk and Bont, 2023).

RSV belongs to the genus Orthopneumovirus of the family Pneumoviridae, with a single serotype and two antigenic subtypes (A and B). It is an enveloped virus with a non-segmented negative-sense RNA genome, causing severe respiratory diseases in infants, the elderly, and immunocompromised individuals (Rima et al., 2017; Chanock and Finberg, 1957).

RSV is a highly contagious virus and is one of the primary causes of severe respiratory infections in infants and young children, accounting for over 30 million cases globally each year, with approximately 10% of these requiring hospitalization (Nair et al., 2010). Clinically, RSV infection usually manifests as an illness of the upper respiratory tract, with symptoms including a runny nose, nasal blockage, coughing, and sneezing, sometimes accompanied by fever and muscle pain. In youngsters than two years old, bronchiolitis and diseases affecting the lower respiratory tract can also be caused by RSV, often associated with small airway obstruction (Muñoz-Escalante et al., 2019). In severe cases, the infection may progress to pneumonia, respiratory failure, apnea, or even death (Agoti et al., 2014). Among adults, RSV has also been identified as a trigger for hospitalizations due to various clinical syndromes, including pneumonia (11%), chronic obstructive pulmonary disease (11%), asthma (7%), and congestive heart failure (5%) (Falsey et al., 2005). Like other RNA viruses including the influenza virus and coronaviruses, RSV exhibits high genetic variability attributed to the absence of proofreading capabilities in its RNA-dependent RNA polymerase. This results in high mutation rates for RSV-A and RSV-B, estimated at 1.48 × 10^-^³ and 1.92 × 10^-^³ substitutions per site annually, respectively (Yu et al., 2021). Consequently, RSV rapidly accumulates single nucleotide polymorphisms (SNPs) and other mutations, leading to continuous genetic and antigenic drift. This high mutation rate significantly undermines the efficacy of vaccines, antiviral drugs, and monoclonal antibodies developed against RSV (Bohmwald et al., 2016).

Because RSV cannot be clinically distinguished from other co-circulating respiratory pathogens based on signs and symptoms alone, it is necessary to conduct laboratory tests on respiratory secretions to verify the presence of infection. Compared to infants, adults tend to have lower viral loads and shorter durations of viral shedding, which limits the sensitivity of current diagnostic methods for detecting RSV in adult patients. Currently, four main diagnostic approaches are available for RSV detection: viral culture, rapid antigen detection tests (RADTs), immunofluorescence (IF) assays, and reverse transcription quantitative real-time PCR (RT-qPCR). Viral culture, once considered the gold standard, is impractical in clinical settings due to its long turnaround time (5–7 days) and low sensitivity (<50%) (Henrickson and Hall, 2007). RADTs and IF assays have replaced culture in routine diagnostics but suffer from low sensitivity (50%-80%) and subjective result interpretation (Chartrand et al., 2015). RT-qPCR, the current gold standard, offers high sensitivity but requires specialized equipment, trained personnel, and high costs, limiting its application in resource-limited settings (Kuypers et al., 2004). RT-qPCR can identify asymptomatic infected individuals, which is an advantage; however, in clinical practice, distinguishing recent infections (requiring intervention) from residual viral shedding (no intervention needed) remains challenging. Approximately 15%-20% of RT-qPCR-positive samples come from individuals with no evidence of acute infection (Lemanske et al., 2005; Kusel et al., 2006; Bulkow et al., 2012), which may affect timely clinical decision-making.

Reverse transcription recombinase-aided amplification (RT-RAA) is an innovative isothermal detection technique that functions at a stable temperature and utilizes four enzymes: recombinase (UvsX, UvsY), single-stranded DNA-binding protein (SSB), and DNA polymerase (Zhang et al., 2017). The underlying principle involves the formation of a complex between UvsX, UvsY, and oligonucleotide primers, which then searches for homologous sequences. Once a homologous region is identified, the corresponding double-stranded DNA is displaced into single strands, allowing the primers to anneal to the complementary sequence. SSB then binds to the resulting single-stranded DNA, and DNA polymerase binds to the primer-DNA complex to initiate amplification. This method is carried out at a stable temperature ranging from 37 to 42 °C, thereby negating the necessity for intricate thermal cycling procedures. As a result, the assay time is significantly reduced and can be completed within 30 minutes. Real-time monitoring of detection results can be achieved with portable fluorescence detectors or blue light emitters. Owing to its exceptional sensitivity, specificity, and rapid response time, RT-RAA has found extensive use in identifying microbial pathogens, establishing itself as a strong asset for point-of-care testing (POCT) (Bai et al., 2020; Wang et al., 2020a, Wang et al., 2020c; Xiong et al., 2020).

In this study, we sought to create an RNA extraction-free RT-RAA assay combined with real-time fluorescence detection for the rapid and convenient identification of RSV. By conducting a comparative analysis with RT-qPCR and RADTs, we assessed the detection capabilities of the RT-RAA approach. Our results demonstrated that the detection time of the RT-RAA assay is similar to that of RADTs, while maintaining the high sensitivity characteristic of RT-qPCR. In summary, this simple, portable, visual, and highly sensitive and specific RSV detection method does not require large or complex equipment, making it highly appropriate for point-of-care applications. This method holds significant promise for practical implementation in the prevention and management of diseases related to RSV.

## 2 Materials and methods

### 2.1 Virus and clinical sample sources

In this research, laboratory-preserved RSV strains were used. In 2025, a total of 265 clinical specimens suspected of being infected with RSV were gathered from Hebei Province, including nasopharyngeal swabs, oropharyngeal swabs, and sputum samples. The samples covered both pediatric (<18 years) and adult (≥18 years) populations, with detailed information on age, gender, clinical symptoms, and epidemiological history provided in **Supplementary Table S1**. Inclusion and exclusion criteria: Samples were included if they met at least one of the following criteria: Acute respiratory infection symptoms (e.g., fever, cough) lasting ≤7 days; Imaging findings suggestive of bronchitis/pneumonia; Epidemiological history (contact with confirmed RSV cases); Clinical suspicion of viral respiratory infection (with exclusion of evidence of bacterial infection). All samples were immediately stored at -80 °C in an ultra-low temperature freezer until further testing.

### 2.2 Design of RT-RAA primers and probe

The M gene sequence of RSV (GenBank accession No. OP242457.1) was down-loaded from the GenBank database and subjected to sequence alignment analysis using DNA STAR software to identify conserved regions. Primers and probes were created utilizing SnapGene software, and the best combinations of primers and probes were chosen based on initial screening techniques. The synthesis of the primers and probes was carried out by Shanghai Biotechnology Co., Ltd. Their sequences and positions are listed in **Table 1**.

**Table 1:** The primers and probes were used in RSV real-time RT-RAA assays.

Primers/Probe	Sequences (5’→3’)	Position
RSV-F1	GACCTTCACTAAGAGTCATGATAAACTCAA	200-299
RSV-F2	AACTCAAGAAGTGCAGTGCTAGCACAAATG	223-252
RSV-F3	AATGCCCAGCAAATTTACCATATGCGCTAA	235-264
RSV-F4	GCAGTGCTAGCACAAATGCCCAGCAAATTT	249-278
RSV-F5	ATGCCCAGCAAATTTACATATGCGCTAATGT	250-281
RSV-F6	CCCAGCAAATTTACCATATGCGCTAATGTG	253-282
RSV-R1	AGGGTTGAGTGTCTTCATAGTGAGATCTTT	388-417
RSV-R2	AATATCATGTGTAGGGTTGAGTGTCTTCAT	400-429
RSV-R3	GTTTTCAAATTCACATAAAGCAATAATATCA	423-453
RSV-R4	GTTTTCAAATTCACATAAAGCAATAATATC	424-453
RSV-R5	TGTTTTCAAATTCACATAAAGCAATAATAT	430-459
RSV-R6	ATGTGAATTTGAAAACATAGTAACATC-AAA	438-467
RSV-probe	AACACACCCTGTGAAATCAAGGCATGTAG(FAM-dT) (THF) (BHQ1-dT) AACATGCCTAAAATC [C3-spacer]	318-365

“Position” indicates the nucleotide location in the RSV M gene (GenBank OP242457.1), oriented 5’→3’.

### 2.3 Nucleic acid extraction

Nucleic acids were obtained from the 265 collected clinical samples following the guidelines of the Qiagen RNA Isolation Kit (Hilden, Germany). During the extraction process, RNase-free water was used as a negative control. All isolated nucleic acids were preserved at -80°C for no longer than 24 hours before further analysis.

### 2.4 Pyrolysis (RNA extraction-free) of clinical samples

The 265 collected clinical samples were processed according to the instructions of the Rapid Nucleic Acid Releaser (RNA)-Type II Kit (AmpFuture, Changzhou, China). RNase-free water was used as the negative control. All extracted nucleic acids were stored at -80 °C for no longer than 24 hours before further analysis.

### 2.5 RT-RAA amplification

The RNA constant-temperature rapid amplification kit (#WLRE8208KIT) was purchased from AmpFuture Biotechnology Co., Ltd. (Weifang, China). Following the guidelines provided in the kit, a reaction mixture of 25 μL was formulated. This mixture included 14.7 μL Buffer A, 4.75 μL RNase-free water, 1.0 μL forward primer (10 μM), 1.0 μL reverse primer (10 μM), 0.3 μL probe (10 μM), 2.0 μL nucleic acid template, and 1.25 μL Buffer B. The reaction tubes were placed in a 7500 Real-time PCR System (Applied Biosystems) and incubated at a temperature of 42 °C for 30 minutes, cycling once per minute while real-time monitoring of fluorescence signals occurred. Simultaneously, the RT-RAA amplification products were visually observed using a portable blue light imaging device (TGreen, Tiangen Biotech Co., Ltd., Beijing, China) with an excitation wavelength of 480 nm.

### 2.6 RT-qPCR detection

Detection via RT-qPCR was conducted using the One Step PrimeScript III RT-qPCR Mix (RR600A, Takara), adhering to the guidelines provided by the manufacturer. The reaction mixture, totaling 25 μL, included 12.5 μL of 2× One Step U+ Mix, 1.5 μL of One Step U+ Enzyme Mix, 1.0 μL of forward primer (10 μM), 1.0 μL of reverse primer (10 μM), 0.5 μL of probe (10 μM), 2.0 μL of nucleic acid template, and 6.5 μL of RNase-free water. This reaction was executed on a 7500 Real-time PCR System (Applied Biosystems) following these cycling parameters: reverse transcription at 55°C for 15 minutes, an initial denaturation step at 95°C for 30 seconds, and subsequently 40 cycles at 95°C for 10 seconds and 60°C for 30 seconds, while continuously monitoring the real-time fluorescence signal.

### 2.7 Rapid antigen detection tests

RADTs were performed using a commercially available colloidal gold-based RSV antigen detection kit (CRIUS: H940-000013) according to the manufacturer’s instructions. Briefly, 100 μL of sample lysate was added to the test cassette, and results were read visually within 15 minutes.

### 2.8 Specificity analysis

Nucleic acids from the following pathogens were utilized as templates to assess the specificity of the assay: Influenza A H1N1, Influenza A H3N2, Influenza A H9N2, Influenza B Victoria lineage, Influenza B Yamagata lineage, Klebsiella pneumoniae, Parainfluenza virus, Rhinovirus, Adenovirus, Human metapneumovirus, Streptococcus pneumoniae, Chlamydia pneumoniae, and Mycoplasma pneumoniae. The assay’s specificity was confirmed through real-time fluorescent RT-RAA amplification employing the chosen specific primers and probe. All pathogen nucleic acids were obtained from Hebei Houqi Biotechnology Co., Ltd. (Baoding, Hebei, China).

### 2.9 Sensitivity analysis

Sensitivity analysis was performed using the laboratory-preserved RSV M gene plasmid (GenBank accession No. OP242457.1) (pMD18-T-M). The plasmid underwent a serial 10-fold dilution to create template concentrations that varied from 105 to 100 copies in a 2 μL volume. A volume of 2 μL from each dilution was used to evaluate the sensitivity of the real-time RT-RAA assay, which was conducted in parallel with the RT-qPCR method. To accurately determine the detection limits, both assays were independently repeated eight times. Probit regression analysis was executed using SPSS software (version 22.0).

### 2.10 Reproducibility and stability analysis

High, medium, and low concentrations of RSV M gene plasmid (GenBank accession No. OP242457.1) (107, 105, and 103 copies/reaction) and clinical sample nucleic acids were selected for testing. Each concentration was tested in triplicate within the same batch and in three independent experiments conducted at different times. To assess the intra-batch and inter-batch reproducibility of the RT-RAA assay, the threshold time’s coefficient of variation (CV) was computed.

### 2.11 Clinical sample testing

A total of 265 clinical samples were tested using the real-time RT-RAA method in parallel with the RT-qPCR assay and RADTs. The concordance between the two methods’ results was compared and analyzed.

### 2.12 Statistical analysis

Probit regression analysis was conducted with SPSS software, utilizing a confidence level of 95% to establish the detection limit. To assess the agreement between the results obtained from real-time RT-RAA, RT-qPCR and RADTs, the Kappa statistic was employed. Chi-square tests were used to compare the sensitivity of RT-RAA and RADTs, with P<0.05 considered statistically significant.

## 3 Results

### 3.1 Optimal primer screening for RT-RAA

**Figure 1:**
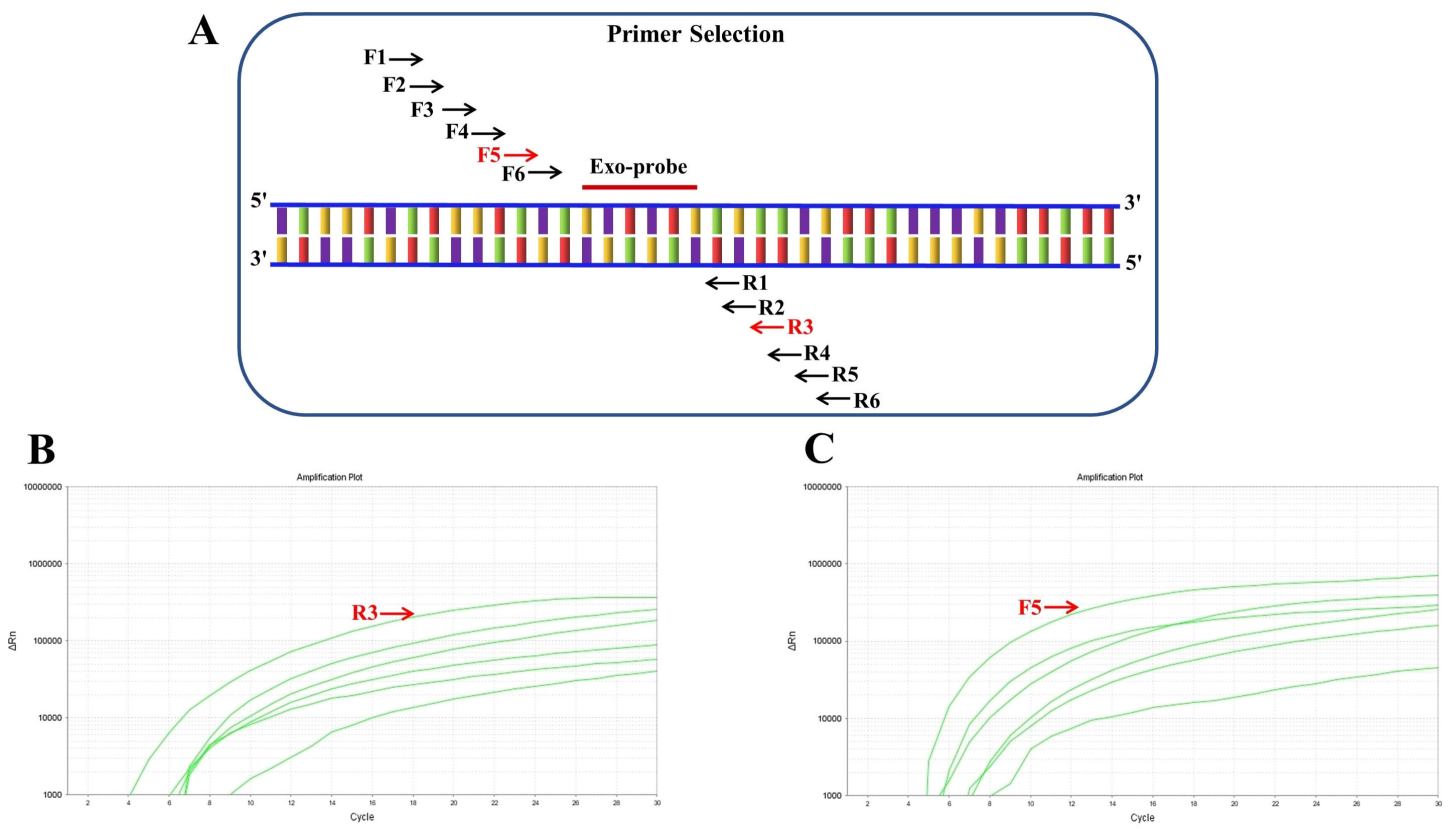
Screening of RT-RAA primers for respiratory syncytial virus detection. **(A)** Schematic representation of the positions of six upstream candidate primers (F1-F6) and six downstream candidate primers (R1-R6) designed based on the Exo-probe. **(B)** Screening results of randomly paired upstream primer F1 with six downstream primers, showing the most significant amplification when paired with R3. **(C)** Screening results using R3 as the fixed downstream primer paired with six upstream primers, indicating that the F5/R3 pair yields the best amplification efficiency.

An Exo-probe targeting positions 318–365 was first designed (**Figure 1A**), based on which six upstream candidate primers (F1-F6) and six downstream candidate primers (R1-R6) were generated (**Figure 1A**). To begin the screening process, the upstream primer F1 was chosen at random and combined with every one of the six downstream primers. The results showed that the F1/R3 pair exhibited the most robust amplification (**Figure 1B**). Subsequently, using R3 as the fixed downstream primer, all six upstream primers were evaluated in various combinations. Among these, the F5/R3 pair demonstrated the best amplification performance (**Figure 1C**). Therefore, the primer pair F5/R3, along with the Exo-probe (targeting positions 318-365), was ultimately selected for real-time RT-RAA detection.

### 3.2 Specific analysis

**Figure 2:**
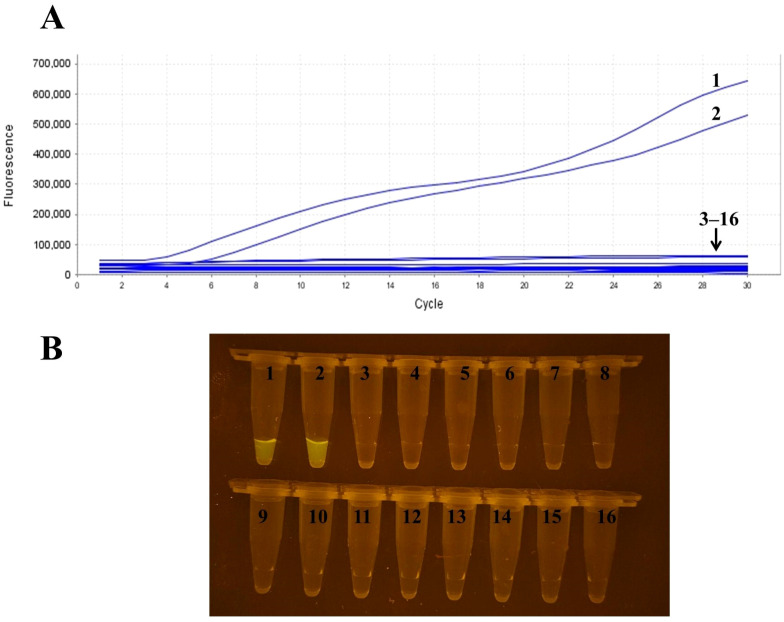
Specificity analysis of RSV RT-RAA detection. **(A)** Real-time fluorescence detection results showing positive amplification only in RSV samples (RSV-A, RSV-B), while other virus samples including influenza A H1N1, H3N2, etc., and the negative control group showed no amplification. **(B)** Visualization of RT-RAA amplification products under a portable blue light de-vice, further confirming the high specificity of the method for RSV. Sample numbers correspond to different templates: samples 1–2 are RSV, samples 3–15 represent influenza A H1N1, H3N2, H9N2, influenza B Victoria lineage, influenza B Yamagata lineage, Klebsiella pneumoniae, parainfluenza virus, rhinovirus, adenovirus, human metapneumovirus, Streptococcus pneumoniae, Chlamydia pneumoniae, and Mycoplasma pneumoniae, respectively; sample 16 is the negative control.

The specificity analysis results demonstrated that only RSV samples (RSV-A and RSV-B) exhibited positive reactions, while samples of H1N1 influenza A, H3N2 influenza A, H9N2 influenza A, Victoria and Yamagata strains of influenza B, Klebsiella pneumoniae, parainfluenza virus, rhinovirus, adenovirus, human metapneumovirus, Streptococcus pneumoniae, Chlamydia pneumoniae, Mycoplasma pneumoniae, and the negative control group all tested negative (**Figure 2A**). Additionally, the products resulting from the RT-RAA assay were directly visible using a portable blue light device, further validating the high specificity of this technique for detecting RSV (**Figure 2B**).

### 3.3 Sensitivity analysis

**Figure 3:**
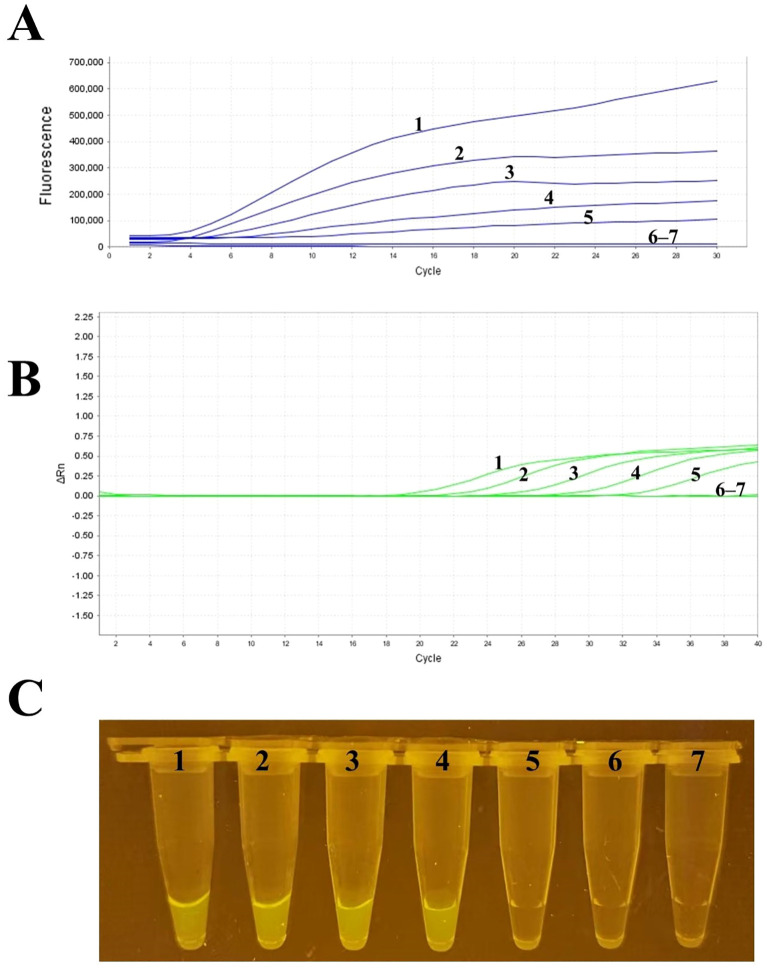
Sensitivity analysis of RSV RT-RAA detection. samples 1–6 in **(C)** represent RSV plasmid templates with 10^5^, 10^4^, 10^3^, 10^2^, 10^1^, and 10^0^ copies per reaction, respectively; sample 7 is the negative control (RNase-free water). All dilutions were prepared with TE buffer (pH 8.0) and calibrated by RT-qPCR. **(A)** Real-time fluorescence readout of RT-RAA assays using serial dilutions of RSV plasmid templates, showing fluorescence signal changes at different template concentrations. The detection limit of RT-RAA was determined to be 159 copies per reaction at a 95% confidence interval. **(B)** Real-time fluorescence readout of RT-qPCR assays using the same serially diluted RSV plasmid templates for sensitivity comparison. **(C)** Visualization results of RT-RAA, indicating a detection limit of 1177 copies per reaction. The different curves or sample numbers correspond to varying template concentrations, with samples 1–6 representing RSV plasmid templates from 10^5^ to 10^0^ copies, and sample 7 as the negative control (RNase-free water). All dilutions were prepared with TE buffer (pH 8.0) and calibrated by RT-qPCR.

The evaluation of the sensitivity for RT-RAA and RT-qPCR was conducted utilizing serial dilutions of plasmid templates containing the RSV M gene. Based on re-al-time fluorescence readouts from both methods, the limit of detection (LOD) for RT-RAA was established at 159 copies per reaction at a 95% confidence interval (**Figures 3A, B**). For visual detection using RT-RAA, the LOD was 1177 copies per reaction (**Figure 3C**). In comparison, for RT-qPCR, the LOD was determined to be 140 copies per reaction.

### 3.4 Repeatability and stability analysis

Reproducibility testing was performed using RSV M gene plasmids and clinical sample nucleic acids at different concentrations. The intra-assay coefficients of variation (CVs) were 4.07%, 4.63%, and 4.61%, while the inter-assay CVs were 4.69%, 5.45%, and 4.27%. All intra- and inter-assay CVs were below 5.66%, indicating that the RNA extraction-free RT-RAA method possesses good repeatability and stability (**Table 2**).

**Table 2:** Results of repeatability and reproducibility detected by real-time RT-RAA assay.

Plasmids concentration	Repeatability (Intra-batch assay)			Reproducibility (Inter-batch assay)		
	Mean	SD	CV (%)	Mean	SD	CV (%)
High (10^7^)	135.33	5.51	4.07	138.67	6.51	4.69
Medium (10^5^)	249	11.53	4.63	253.33	13.80	5.45
Low (10^3^)	379.67	17.50	4.61	380.33	16.26	4.27
RSV nucleic acid	279.33	10.07	3.60	277.33	15.70	5.66

“Mean” refers to the average threshold time (Tt) in minutes; “SD” = standard deviation; “CV” = coefficient of variation.

### 3.5 Clinical sample testing

A total of 265 clinical samples were assessed utilizing both real-time RT-RAA, RT-qPCR and RADTs. The real-time fluorescence-based RT-RAA assay identified 112 positive samples, which were in complete agreement with the RT-qPCR results. The real-time RT-RAA method exhibited a sensitivity of 100%, specificity of 100%, and overall accuracy of 100% (265/265), with a Kappa value of 1, indicating excellent concordance between the two methods. In comparison, RADTs detected 105 positive samples among the 112 RT-qPCR-positive samples, showing a sensitivity of 93.8% (105/112) and a specificity of 89.5% (137/153 negative samples), with a Kappa value of 0.820. These results confirm that RT-RAA is significantly more sensitive than RADTs. Additionally, among the 112 samples that tested positive via real-time RT-RAA, 107 were concurrently classified as positive through visual RT-RAA detection. The visual RT-RAA assay showed a sensitivity of 95.5% (107/112), specificity of 100%, and an overall accuracy of approximately 98.5% [(107 + 153)/265], with a Kappa value of 0.961.

Five samples that were positive by real-time RT-RAA but negative by visual detection had RT-qPCR CT values indicating viral loads close to the visual detection limit: Sample S089 (CT = 34.2, ~1300 copies/reaction), S156 (CT = 35.5, ~1250 copies/reaction), S192 (CT = 36.1, ~1200 copies/reaction), S215 (CT = 36.3, ~1190 copies/reaction), and S253 (CT = 37.8, ~1180 copies/reaction). These results further confirm the strong correlation between the two detection approaches (**Table 3**).

**Table 3:** Comparison of the RSV real-time RT-RAA with the RT-qPCR assay and RADTs on clinical samples.

Assay		RT-qPCR		Sensitivity	Specificity	Kappa
		Positive	Negative			
Real-time RT-RAA (Via real-time fluorescence read-out)	Positive	112	0	100%	100%	1
	Negative	0	153			
	Total (265)	112	153			
Real-time RT-RAA (Via visual detection)	Positive	107	0	95.5%	100%	0.961
	Negative	5	153			
	Total (265)	112	153			
RADTs	Positive	105	16			
	Negative	7	137	93.8%	89.5%	0.820
	Total (265)	112	153			

To confirm the specificity of RT-RAA amplification, 20 positive products (covering high, medium, and low viral loads) were randomly selected for Sanger sequencing. The sequences showed 100% identity with the RSV M gene (GenBank OP242457.1), with no non-specific amplification.

## 4 Discussion

In 1956, Morris and colleagues first identified this pathogen while studying acute respiratory disease in chimpanzees. It was officially named respiratory syncytial virus (RSV) in 1957 due to its distinctive cytopathic effects. RSV accounts for the primary reason behind acute respiratory infections that lead to outpatient consultations, emergency room visits, and hospital admissions for children globally under the age of five. During seasonal outbreaks, the number of RSV infections often exceeds those caused by influenza and other viruses. The epidemic season typically spans from autumn to spring, with cases emerging earlier in temperate regions (Hall et al., 2009; Papenburg et al., 2012; Zhou et al., 2012). Preterm infants and immunocompromised individuals are at an elevated risk for serious infections (Papenburg and Boivin, 2010), while RSV notably affects the rates of morbidity and mortality among adults with pre-existing health conditions and the elderly demographic (Falsey et al., 2005). RSV is transmitted both through direct contact with respiratory droplets released by coughing or sneezing patients and indirectly via contact with contaminated secretions on environmental surfaces. Given the absence of distinctive clinical signs and symptoms that set RSV infection apart from other simultaneously circulating respiratory pathogens, a precise laboratory diagnosis of RSV in respiratory samples is essential. Currently, treatment for RSV-associated acute respiratory infections mainly relies on supportive care. Early and precise diagnosis can reduce empirical antibiotic use and allow timely adjustment of therapy in patients already receiving antibiotics, thereby avoiding unnecessary medication—an especially important consideration for infants with severe bacterial coinfections (Adcock et al., 1997; Woo et al., 1997; Barenfanger et al., 2000; Byington et al., 2002; Purcell and Fergie, 2002; Ferronato et al., 2012; Rogers et al., 2015). Moreover, RSV is a common pathogen responsible for nosocomial outbreaks. Rapid detection facilitates timely isolation measures to interrupt transmission chains, optimize bed management, and improve both infection control efficiency and healthcare quality (Madge et al., 1992; Mills et al., 2011).

The identification of RSV through microbiological diagnosis mainly relies on successfully detecting the virus in respiratory secretions from individuals. Prior to the development of innovative rapid molecular diagnostic tests, four main diagnostic methods were commonly used: cell culture, RT-qPCR, IF, and RADTs. Cell culture was previously deemed the gold standard due to its high specificity, but it suffers from low sensitivity, complex procedures, and lengthy turnaround times. RT-qPCR has become the new gold standard because of its high sensitivity, specificity, and rapid turnaround; however, it requires specialized personnel and equipment, making it primarily suitable for large centralized laboratories. Additionally, interpreting RT-qPCR results can be challenging—for example, prolonged viral shedding in recovered patients may lead to false-positive interpretations. IF offers a relatively quick diagnostic option but has lower sensitivity compared to RT-qPCR and relies on skilled personnel and specific equipment. Traditional RADTs depend on visual interpretation of results, which can be subjective, and their relatively low sensitivity makes them unreliable for ruling out RSV cases during outbreaks. Serological testing, while capable of identifying infections based on antibody titers, is limited in its application since some children do not undergo seroconversion after infection. With advancements in molecular biology, novel automated immunoassays and molecular diagnostic methods have emerged. Although these techniques offer standardized result interpretation, they require auxiliary equipment and are costly, making them unsuitable for point-of-care or large-scale screening (Queiróz et al., 2002; Henrickson and Hall, 2007).

In recent years, nucleic acid isothermal amplification technologies have become essential tools for detecting RNA viruses. This category includes various techniques such as loop-mediated isothermal amplification (LAMP) (Fukuta et al., 2003; Meyers et al., 2023), recombinase polymerase amplification (RPA) (Zaghloul, 2014; Lobato and O’Sullivan, 2018), recombinase-aided amplification (RAA) (Chen et al., 2018), nucleic acid sequence-based amplification (NASBA) (Gabrielle et al., 1993; Li et al., 2023a), and helicase-dependent amplification (HDA) (Goldmeyer et al., 2007; Zasada et al., 2022). By amplifying DNA at a constant temperature, these methods eliminate the need for the thermal cycling required by PCR. Due to their simple equipment requirements, rapid detection times, and high feasibility, isothermal amplification techniques are well-suited for point-of-care testing and resource-limited settings (Obande and Banga Singh, 2020). As a novel isothermal amplification method, RT-RAA utilizes DNA polymerase, UvsX, UvsY and SSB to complete amplification within 30 minutes at a constant temperature of 37-42°C (Yan et al., 2018; Li et al., 2023c, Li et al., 2023b). It requires only a single primer pair, making it more efficient than LAMP, which demands 4–6 primers and operates at 65°C (Pang and Long, 2023). When combined with portable devices such as lateral flow devices (LFDs), handheld isothermal fluorescence detectors, or portable blue-light illuminators, RT-RAA effectively meets the needs of on-site detection (Chen et al., 2021; Cui et al., 2022; Li et al., 2023c). It has been widely applied across various pathogen detection scenarios, covering animal, human, and animal-derived food sectors (Wang et al., 2020b; Ding et al., 2022; Feng et al., 2022; Xia et al., 2022, Xia et al., 2024; Li et al., 2024).

The RNA extraction-free RT-RAA method established in this study demonstrated significant advantages in RSV detection, showing better compatibility and innovation compared to previously reported methods. Compared with PCR or RT-qPCR diagnostic methods that rely on purified nucleic acids, our RAA assay eliminates the need for conventional nucleic acid extraction, reducing sample preparation time from 2 hours to just 5 minutes. From a technical perspective, this method is similar to the multiplex RT-RAP technology developed by Fan et al (Fan et al., 2023), enabling rapid nucleic acid amplification at a constant temperature of 42 °C without the thermal cycling required in conventional PCR, thereby simplifying the workflow and reducing the detection time to within 30 minutes. Regarding detection performance, the RT-RAA developed here achieved a detection limit of 159 copies/reaction for RSV (95% confidence interval), surpassing the sensitivity of 162 copies/reaction reported by Zhou et al (Zhou et al., 2025). Notably, this study introduced a visual detection mode, which, although with a detection limit of 1177 copies/reaction, offers practical utility in resource-limited settings through the use of a portable blue-light device. This aligns well with the point-of-care testing requirements emphasized by Hou et al. in bovine RSV detection (Qi et al., 2019). Clinically, the detection results of 265 samples by RT-RAA were in complete agreement with RT-qPCR (Kappa = 1), corroborating the 100% concordance reported by Fan et al. in 252 clinical samples, thus confirming the clinical re-liability of the RT-RAA assay. Moreover, RT-RAA showed significantly higher sensitivity than RADTs (100% *vs*. 93.8%), addressing the limitation of low sensitivity in traditional antigen-based tests. The establishment of the RT-RAA assay critically depends on the design of primers and probes; the optimal primer pair identified in this study (R3/F5) exhibited high specificity with no cross-reactivity observed against 13 common respiratory pathogens including influenza A/B viruses and Klebsiella pneumoniae. Sequencing verification further confirmed the specificity of the amplified products. Overall, streamlining the procedure reduces the likelihood of errors and minimizing reagent use lowers costs, which facilitates the promotion and clinical application of this assay. Building on the strengths of existing RAA technology, this study further enhanced the field applicability of RSV detection by integrating an RNA extraction-free strategy, optimizing the target gene, and incorporating visual detection. The performance of this method complements similar approaches reported in the literature, providing a diversified technical solution for rapid diagnosis of respiratory viruses.

In summary, the RSV RNA extraction-free RT-RAA assay developed in this study is characterized by high sensitivity, accuracy, rapidity, ease of operation, and cost-effectiveness, making it highly suitable for point-of-care testing. This method provides robust technical support for early surveillance and precise control of RSV infections. It is portable, requires no bulky instrumentation, and enables visual detection under blue light, while maintaining excellent sensitivity and specificity. Clinical evaluation demonstrated 100% concordance with RT-qPCR results, fully validating its re-liability and potential for clinical application.

## Data Availability

The original contributions presented in the study are included in the article/**Supplementary Material**. Further inquiries can be directed to the corresponding authors.
